# Trial protocol for COLO‐DETECT: A randomized controlled trial of lesion detection comparing colonoscopy assisted by the GI Genius™ artificial intelligence endoscopy module with standard colonoscopy

**DOI:** 10.1111/codi.16219

**Published:** 2022-06-28

**Authors:** Alexander Seager, Linda Sharp, James S. Hampton, Laura J. Neilson, Tom J. W. Lee, Andrew Brand, Rachel Evans, Luke Vale, John Whelpton, Colin J. Rees

**Affiliations:** ^1^ South Tyneside and Sunderland NHS Foundation Trust South Tyneside District Hospital, South Shields Tyne and Wear UK; ^2^ Newcastle University—Population Health Sciences Institute Newcastle University Centre for Cancer Newcastle Upon Tyne UK; ^3^ Northumbria Healthcare NHS Foundation Trust North Tyneside General Hospital, North Shields UK; ^4^ North Wales Organisation for Randomised Trials in Health (NWORTH) Bangor UK; ^5^ Newcastle University—Health Economics Group, Population Health Sciences Institute Newcastle University Centre for Cancer Newcastle Upon Tyne UK; ^6^ Patient and Participant Involvement Representative Newcastle University‐Population Health Sciences Institute, Newcastle University Centre for Cancer Newcastle Upon Tyne UK

**Keywords:** adenoma, artificial intelligence, colonoscopy, colorectal cancer, computer‐aided detection, economic evaluation

## Abstract

**Aim:**

Colorectal cancer is the second commonest cause of cancer death worldwide. Colonoscopy plays a key role in the control of colorectal cancer and, in that regard, maximizing detection (and removal) of pre‐cancerous adenomas at colonoscopy is imperative. GI Genius™ (Medtronic Ltd) is a computer‐aided detection system that integrates with existing endoscopy systems and improves adenoma detection during colonoscopy. COLO‐DETECT aims to assess the clinical and cost effectiveness of GI Genius™ in UK routine colonoscopy practice.

**Methods and analysis:**

Participants will be recruited from patients attending for colonoscopy at National Health Service sites in England, for clinical symptoms, surveillance or within the national Bowel Cancer Screening Programme. Randomization will involve a 1:1 allocation ratio (GI Genius™‐assisted colonoscopy:standard colonoscopy) and will be stratified by age category (<60 years, 60–<74 years, ≥74 years), sex, hospital site and indication for colonoscopy. Demographic data, procedural data, histology and post‐procedure patient experience and quality of life will be recorded. COLO‐DETECT is designed and powered to detect clinically meaningful differences in mean adenomas per procedure and adenoma detection rate between GI Genius™‐assisted colonoscopy and standard colonoscopy groups. The study will close when 1828 participants have had a complete colonoscopy. An economic evaluation will be conducted from the perspective of the National Health Service. A patient and public representative is contributing to all stages of the trial. Registered at ClinicalTrials.gov (NCT04723758) and ISRCTN (10451355).

**What will this trial add to the literature?:**

COLO‐DETECT will be the first multi‐centre randomized controlled trial evaluating GI Genius™ in real world colonoscopy practice and will, uniquely, evaluate both clinical and cost effectiveness.

## INTRODUCTION

Colorectal cancer (CRC) is the second commonest cause of cancer death worldwide killing >915 000 per year [1]. In the UK, CRC affects one in 15 men and one in 18 women in their lifetime [2]. Most CRCs develop via the adenoma–carcinoma sequence [3], with mutation of multiple proto‐oncogenes and tumour suppressor genes potentially contributing to malignant transformation [4, 5, 6]. Non‐adenomatous polyps (including sessile serrated lesions and hyperplastic polyps) also play a role, albeit more minor, in CRC development [7, 8]. Carcinoma development may be disrupted, and cancer prevented, by removal of pre‐malignant lesions.

### Post‐colonoscopy CRC and adenoma detection

Colonoscopy is the gold standard investigation to seek colorectal adenomas (or CRC). However, adenomas may be missed due to incomplete mucosal visualization or failure to detect a visible lesion. Tandem colonoscopy (where a patient undergoes back‐to‐back colonoscopies) has estimated the miss rate to be 22% for all polyps, with an inverse relationship of miss rate to polyp size [9]. Missed lesions can result in post‐colonoscopy CRC (PCCRC) [10]—CRC occurring within 3 years of a non‐diagnostic colonoscopy [11]—and small (<10 mm), flat adenomas are over‐represented amongst missed lesions [9, 12]. A low adenoma detection rate (ADR, the proportion of colonoscopies in which one or more adenomas is detected) correlates with a higher rate of PCCRC [12, 13, 14, 15, 16, 17], and each 1% increase in ADR correlates with 3% reduction in PCCRC incidence [14].

### Improving adenoma detection

Multiple interventions have been recommended to improve adenoma detection, including measures to improve technique (e.g., longer withdrawal time, patient position change), improvement of bowel preparation, and devices that attach to the end of the colonoscope to increase mucosal visualization [18, 19, 20, 21, 22]. Attempts to improve the ability to identify lesions on the visualized mucosa include high definition colonoscopes [23], colonoscopes with increased field of view [24] and enhanced image analysis such as virtual chromoendoscopy [25], although an extensive network meta‐analysis suggests that no single strategy or category of assistive technology has a clear advantage [26].

### Computer‐aided detection of colorectal lesions

Technological advances, alongside the finding that a second observer improves adenoma detection [27, 28, 29], have stimulated the development of computer‐aided detection (CADe) systems (essentially a virtual second observer). Most current CADe systems are based on convolutional neural networks [30, 31, 32, 33], a form of deep learning optimal for complex image analysis, in which the video feed from the colonoscope is analysed and processed in real time to generate an output that is useful to the operator [34].

### GI Genius™

**FIGURE 1:**
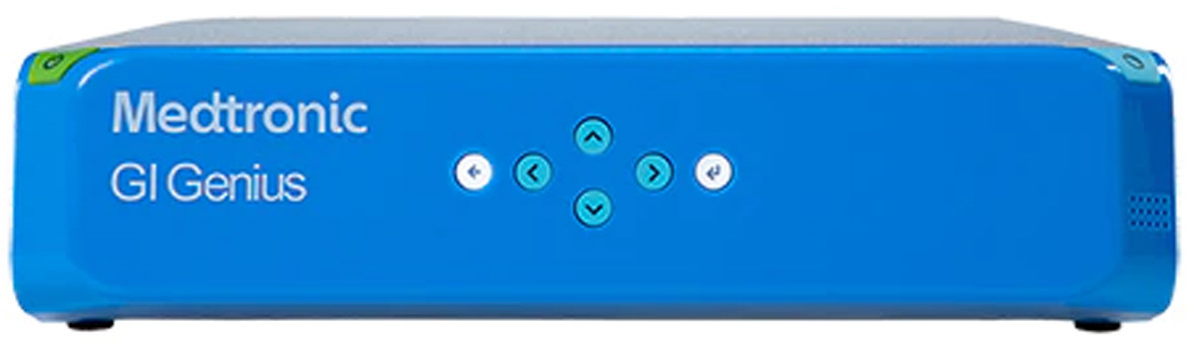
The GI Genius™ intelligent endoscopy module (©Medtronic Ltd)

**FIGURE 2:**
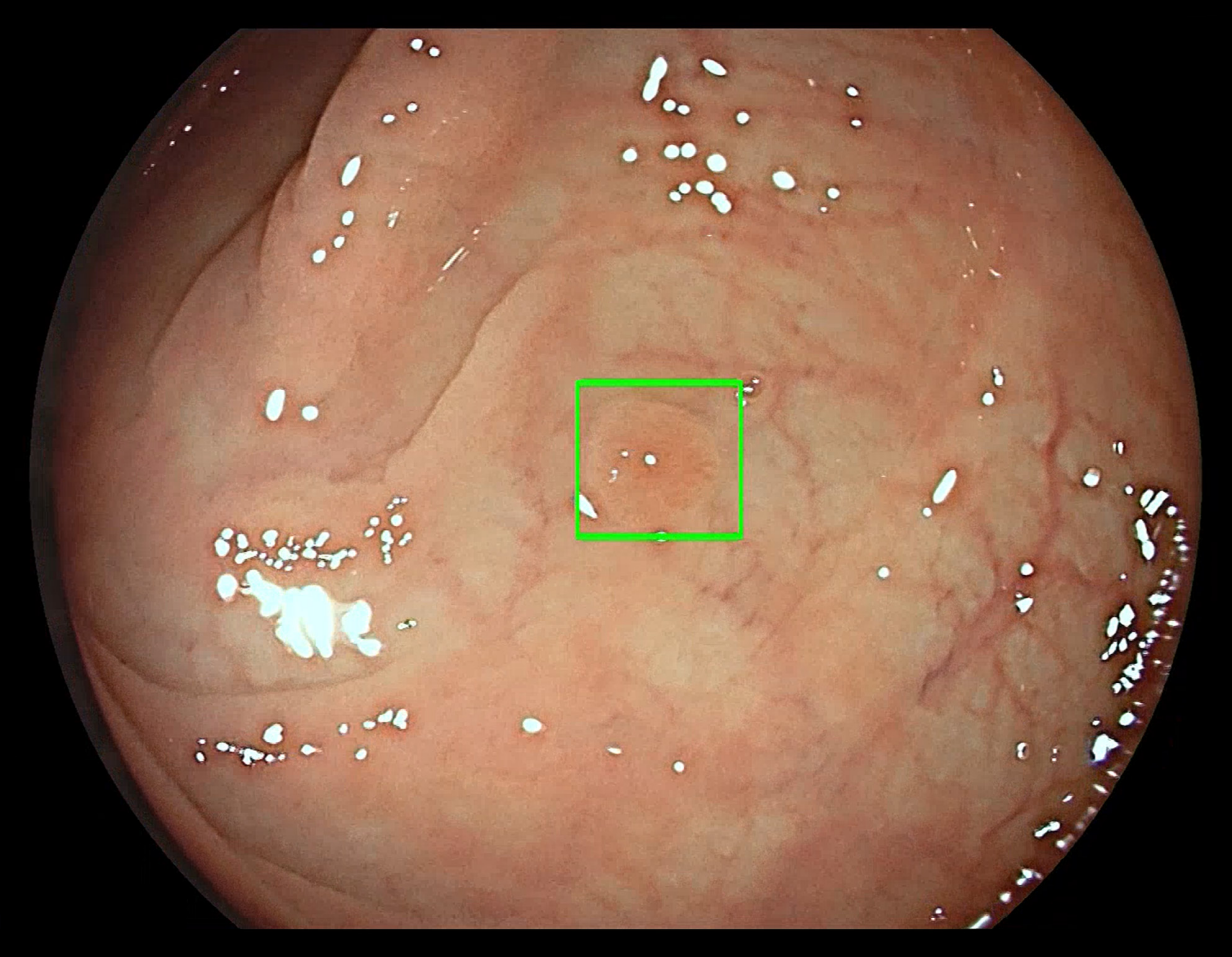
A colonic polyp highlighted within a green bounding box by GI Genius™ (©Medtronic Ltd)

GI Genius™ (Medtronic Ltd) is a convolutional‐neural‐network‐based artificial intelligence module (Figure 1) which integrates with most endoscopy systems from all major manufacturers. It indicates potential abnormalities by projecting a green box on the monitor (Figure 2) and emitting an adjustable‐volume audible alert, without perceptible lag. The colonoscopist may then inspect the highlighted area more closely and decide whether to ignore, biopsy or excise the putative abnormality. The first randomized controlled trial (in expert centres only) found that using GI Genius™ improved the ADR by 14% in patients presenting with gastrointestinal symptoms, undergoing surveillance colonoscopy and participating in a national CRC screening programme [35]. These findings have since been confirmed with non‐expert colonoscopists [36]; although this improves the generalizability of the findings, they were pooled from two separate trials, with no trial having yet studied expert and non‐expert practice simultaneously. Furthermore, no study has yet conducted an economic evaluation of CADe.

### COLO‐DETECT

COLO‐DETECT is a parallel arm, multi‐centre (Box 1) randomized controlled trial that will compare polyp detection during GI Genius™‐assisted colonoscopy (GGC) to detection during standard colonoscopy (SC), in the context of routine colonoscopy practice in the UK. This includes patients referred for reason of gastrointestinal symptoms or requiring surveillance colonoscopy (together henceforth referred to as the ‘symptomatic population’) or as part of the national Bowel Cancer Screening Programme (BCSP, the ‘screening population’). Whilst tandem colonoscopy studies have been used elsewhere in endoscopy studies, and require smaller sample sizes to demonstrate effect, it was felt that a parallel arm design was both more pragmatic and reflective of clinical practice and thus more in keeping with the ethos of the study, especially as the sample size required to demonstrate a significant difference in detection using a parallel arm design was not deemed excessive (see the section Sample size, below).

BOX 1 **List of COLO‐DETECT study sites (subject to potential new sites)**

**Table:** 

South Tyneside and Sunderland NHS Foundation Trust (Sponsor site), North Tees and Hartlepool Hospitals NHS Foundation Trust, South Tees Hospitals NHS Foundation Trust, Kettering General Hospital NHS Foundation Trust, University Hospitals of Morecambe Bay NHS Foundation Trust, Bolton NHS Foundation Trust, Royal Wolverhampton NHS Trust, Newcastle‐upon‐Tyne Hospitals NHS Foundation Trust, Northumbria Healthcare NHS Foundation Trust
NHS, National Health Service.

## METHODS

### Study objectives

The COLO‐DETECT primary, key secondary and other secondary study objectives are listed in Box 2. They represent the intention to thoroughly assess the clinical and cost effectiveness of the GI Genius™.

BOX 2 **COLO‐DETECT study objectives**

**Table:** 

Primary objective: to compare mean number of adenomas per procedure (MAP) between GGC and SC Key secondary objective: to compare ADR between GGC and SC Other secondary objectives are*: to determine whether there is a difference in MAP and/or ADR between GGC and SC within each of the screening and symptomatic populations to determine whether there is a difference in mean polyps per procedure (MPP) or polyp detection rate (PDR) between GGC and SC to ascertain the nature and distribution of all polyps in the colorectum, including location, size and morphology, comparing GGC and SC to ascertain if there is a difference in the detection rate of sessile serrated polyps (SSP) between GGC and SC to ascertain if there is a difference in cancer detection rate between GGC and SC (this includes polyps removed at colonoscopy and subsequently found to be cancerous, and lesions felt to be cancerous at the time of colonoscopy) to determine whether there is a difference in advanced adenoma detection rate (AADR) between GGC and SC to determine if there is a difference in caecal intubation rate, insertion time to caecum, total procedure time, withdrawal time in the absence of polyps, and colonoscopist‐assessed and nurse‐assessed patient comfort scores between GGC and SC to determine if there is a difference in patient experience between GGC and SC, utilizing a validated patient‐reported experience measure [37] to identify any projected difference in future post‐polypectomy surveillance colonoscopy workload, according to the application of national guidelines [38] to participant‐level polyp data comparing GGC and SC to compare MAP and ADR between BCSP and non‐BCSP colonoscopists, comparing GGC and SC to compare MAP and ADR between colonoscopists of differing experience levels, as indicated by lifetime procedure numbers and average annual numbers of procedures performed in the last 3 years to compare MAP and ADR for the first 20% of study participants scoped by each colonoscopist with the last 20% of participants to identify any changes in MAP or ADR throughout the study to compare baseline MAP and ADR of each colonoscopist (6 months pre‐study) with that colonoscopist's MAP and ADR during the study in the SC arm (to assess for a contamination effect) and for 6 months post‐study (to assess for dependence upon GI Genius™) to collect contemporaneous data on MAP and ADR for colonoscopists not taking part in the study to follow up patients to identify future outcomes related to colorectal polyps or CRC In addition, further secondary objectives are: to collect data that will allow subsequent health economic evaluation to determine the cost effectiveness of GGC compared with SC to undertake a process evaluation including investigation of user experience of GI Genius™ (see Box 3)
ADR, adenoma detection rate; BCSP, Bowel Cancer Screening Programme; GGC, GI Genius™‐assisted colonoscopy; SC, standard colonoscopy. *secondary objectives 2‐15 will be assessed across the whole study population and between the symptomatic and screening populations.

The primary and key secondary objectives will be assessed across the whole study population, as well as within the symptomatic and screening populations, and according to colonoscopist experience. Other important markers of detection will be measured (such as polyp detection rate and polyp distribution) as well as procedural factors, patient experience and measures of the impact of GI Genius™ on colonoscopy services (especially post‐polypectomy surveillance burden). Given the noteworthy lack of cost effectiveness analysis for most assistive strategies or technologies, data will be collected to enable the economic evaluation of the cost effectiveness of GI Genius™ to inform decisions concerning wider adoption. Additionally, in recognition that financial issues alone do not determine uptake of novel interventions, a process evaluation will be undertaken to appreciate issues potentially affecting adoption and utilization of the GI Genius™ (see Box 3).

BOX 3 **Description of planned process evaluation**

**Table:** 

Evaluating and adopting novel technology, especially high‐cost technology, is often a lengthy and convoluted process, involving multiple stakeholders with often differing and sometimes conflicting priorities. COLO‐DETECT will include a process evaluation [39] which will seek to understand user experience and fidelity in relation to GI Genius™; identify any moderating contextual factors and/or unintended consequences of using GI Genius™; and explore barriers and facilitators to wide‐scale implementation of the technology into clinical practice. This will include scrutiny of trial data (e.g., deviations from protocol) and semi‐structured interviews with a range of stakeholders including (but not limited to) clinician users of the technology, service managers, healthcare commissioners and guideline‐making bodies such as the British Society for Gastroenterology. This will serve as an exemplar for the process of evaluating and adopting novel technology into the National Health Service (NHS).

### Study process

Potential participants will be identified from colonoscopy referrals according to site‐specific pathways and will usually be approached by telephone to assess interest and eligibility (to minimize face‐to‐face contact in the COVID‐19 era and increase convenience for patients). Inclusion and exclusion criteria are listed in Box 4. If interested and eligible, they will be sent an invitation letter, patient information sheet and consent form.

BOX 4 **COLO‐DETECT inclusion, exclusion and withdrawal criteria**

Inclusion criteria:

- 18 years of age or over
- able to give informed consent
- referred for a colonoscopy for
  - clinical symptoms
  - surveillance after previous colonoscopy or bowel pathology
  - within the BCSP
  - colonoscopy is to be performed by a colonoscopist trained to perform GGC within the trial

Exclusion criteria:

- absolute contraindications to colonoscopy
- confirmed or expected pregnancy
- established or suspected large bowel obstruction or pseudo‐obstruction
- current colorectal cancer or polyposis syndrome
- known colonic stricture (meaning colonoscopy may be incomplete)
- known active colitis (e.g., ulcerative colitis, Crohn's colitis, diverticulitis, infective colitis)
- antiplatelet or anticoagulant therapy (except daily low‐dose [75 mg] aspirin) which has not been stopped for the procedure (precluding polypectomy)

Patients will also be excluded if they are undergoing any of the following:

- inflammatory bowel disease surveillance procedure
- planned therapeutic procedure or assessment of a known lesion
- colonoscopy after referral for polyps identified on a Bowel Scope procedure (Bowel Scope was the national screening programme offering 55–59‐year‐olds a one‐off flexible sigmoidoscopy—it has ceased to be offered but some patients may have outstanding procedures that were delayed due to COVID‐19)

Patients in other research studies are not excluded from recruitment to COLO‐DETECT, other than where the colonoscopy is a follow‐up procedure in an interventional study examining prevention of adenoma or CRC recurrence.

Withdrawal criteria:

- withdrawal of consent to participate in the trial or for the colonoscopy at any point up to or during the procedure
- new diagnosis of a polyposis syndrome
- new diagnosis of active colitis

For any participant who withdraws, data will be collected up to the point of withdrawal, and used.

Upon attending for colonoscopy, consent will be confirmed in writing and participants will complete the EQ‐5D‐5L health‐related quality of life questionnaire [40]. Participants will be randomized by recruiting staff 1:1 to GGC or SC [41] using a secure web‐based platform. Randomization will be stratified by age category (<60 years, between 60 and <74 years, ≥74 years), sex, study site and referral category (symptomatic or screening population). In the event of randomization platform unavailability, a coin toss will be used.

Standard colonoscopy represents standard care for colonoscopy at each study site. GGC incorporates all elements of SC and differs only in that the GI Genius™ will be turned on and activated for at least the whole of the inspection phase of withdrawal, although the investigators recommend to participating colonoscopists that the GI Genius™ is activated prior to the procedure. Procedural data will be recorded onto a Case Report Form (CRF). Post‐procedure, participants in both arms will undergo standard post‐colonoscopy care and discharge procedures for each site.

Prior to discharge participants will be given a patient experience questionnaire (the validated Newcastle ENDOPREM™ [37]) and a second iteration of the EQ‐5D‐5L to complete and return in a pre‐paid envelope. Follow‐up will cease upon completion of a post‐procedure review between days 14 and 21. This may include accessing medical records (including endoscopy reports and histology) and a telephone call to assess whether any adverse events (AEs) or serious adverse events (SAEs) have occurred. No further contact will be required for study participation, although participants will be asked to consent to long‐term (up to 5 years) passive follow‐up of health outcomes through endoscopy data, medical records and national databases such as the National Cancer Registry Database, Hospital Episode Statistics data, National Endoscopy Database [42] and link to the CORECT‐R data repository [43]. Where data are currently anonymized participant consent will be sought to link data when this becomes possible. Present and future clinical care will be unaffected by study participation.

### Colonoscopists and training with GI Genius™

**FIGURE 3:**
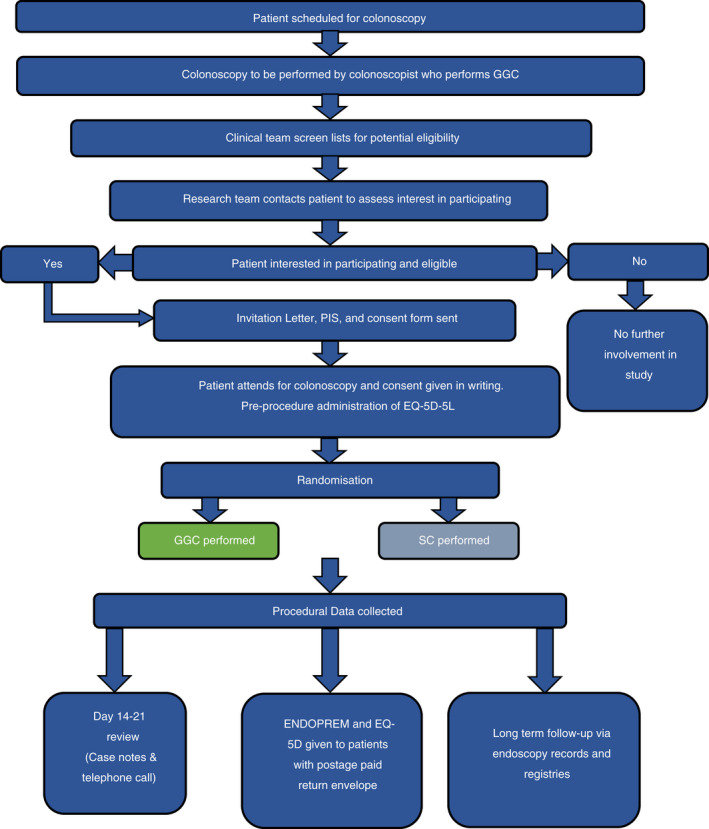
COLO‐DETECT participant flowchart

Every colonoscopist at participating sites will be eligible to participate. Participating colonoscopists will undergo standardized training in how to use the GI Genius™ and to deliver SC and GGC according to study protocol, after which they will be required to perform a set number of GGC procedures prior to participating. No testing will be conducted unless requested by the colonoscopist. Colonoscopists who perform colonoscopy within the BCSP undergo additional accreditation and so may be considered to represent expert practice [15], meaning that over‐representation of BCSP‐accredited colonoscopists may not reflect routine clinical practice in the UK. A range of experience amongst participating colonoscopists is anticipated and desirable; to facilitate subgroup analysis by colonoscopist experience, it will be assessed by BCSP accreditation status, lifetime procedure numbers and yearly procedure numbers for the last 3 years (Figure 3).

### Sample size

The primary outcome for COLO‐DETECT is the difference in MAP between GGC and SC (for which a clinically meaningful mean difference has been considered to be 0.50 for screening patients and 0.30 for symptomatic patients) (based on communications between the senior author, the clinicians involved in the trial and taking opinion from other experts). Due to the importance of ADR in detection studies and its historical widespread use it is important that this study is also powered to demonstrate a difference in ADR.

Exploratory—and, importantly, separate—sample size calculations for MAP and ADR indicate that a larger sample would be required to detect a significant difference in ADR than in MAP; COLO‐DETECT has therefore been powered to detect a clinically meaningful and statistically significant difference in ADR, which exceeds that required to detect such a difference in MAP. This represents a difference of 7% in ADR (with a clinically meaningful difference deemed to be 10% for screening patients and 5% for symptomatic patients). Assuming a mixed sample of 40% screening (BCSP) patients and 60% symptomatic patients, with a baseline ADR of 27.6% across the sample (by aggregating BCSP and non‐BCSP data), a superiority design including 2032 participants will be required to provide 90% power for a two‐sided test for difference in proportions with an alpha value of 0.05. Allowing for 10% attrition due to incomplete colonoscopies, the target sample size will be 1828 participants having a complete colonoscopy. This sample size will more than enable detection of a pooled difference of 0.18 mean difference in MAP.

### Data collection

Baseline data (age, sex, indication for colonoscopy, colonoscopist study ID and BCSP accreditation status) will be recorded into a CRF pre‐procedure along with trial arm. Post‐procedure, the following data will be recorded:

- medications used
- video processor manufacturer
- use of a mucosal visualization device
- procedure timings (start/end times, withdrawal commencement and withdrawal time—in minutes)
- furthest extent reached (and reason caecum not reached, if relevant)
- whether retroflexion was performed
- quality of bowel preparation (using standard JAG classifications)
- nurse‐assessed and colonoscopist‐assessed patient comfort scores (using the modified Gloucester comfort score)
- pathology identified (including polyps, cancer, inflammatory bowel disease etc.)
- whether any polyps were detected on insertion or withdrawal
- polyp location, size, morphology, removal method and retrieval status
- histology of any biopsies or polyps retrieved
- occurrence of any AEs or SAEs
- anticipated interval to any subsequent surveillance colonoscopy (based solely on the application of the latest British Society for Gastroenterology guidelines [38] to recorded endoscopic findings and histology)
- whether GI Genius™ was deactivated or suffered a technical malfunction during the colonoscopy (relevant for planned process evaluation)

If required, participants will receive the following: a verbal reminder (by telephone) from a member of the research team to complete and return their post‐procedure questionnaires; with a written reminder (and another copy of the questionnaire) sent 4 weeks post‐procedure.

### Adverse events

Adverse events (any new medical occurrence or worsening of an existing condition) will be monitored from the date of colonoscopy until cessation of follow‐up or withdrawal from the study (whichever occurs first) and will be recorded on medical notes and CRFs.

Serious adverse events will be managed clinically as per normal procedures. They will be recorded on a bespoke form and reported to the sponsor as soon as the local research team becomes aware of the SAE, in line with current good clinical practice guidance [44]. SAEs must be reported to the sponsor even if they are considered by the local site team and Principal Investigator to be expected, or unrelated to the colonoscopy. If the sponsor deems it necessary, they will be reported to the NHS Research Ethics Committee (REC) within 15 days. Standard NHS complaints procedures and indemnity arrangements are in place to support participants who have been harmed during study participation.

GI Genius™‐assisted colonoscopy is not known to entail any additional risks to participants. The exception would be that, if more polyps are identified during GGC than SC, then assessing and managing these may result in an increase in procedure duration (provisional estimate of 5% additional time), which may affect patient comfort; and if additional polypectomies are performed then the overall risk of complications (mainly bleeding, perforation) may be increased. Universally accepted best practice in colonoscopy remains that all polyps (except rectal hyperplastic polyps) should be excised, assuming that the marginally increased risk of complications does not outweigh the potential benefit of reduced future CRC risk. Accordingly, AEs will be monitored to determine whether any increase in detection leads to more polypectomies and whether this correlates with an increased AE rate, including bleeding and perforation as well as sedation‐related risks. This will be critical for informing the planned economic evaluation.

All AEs and SAEs will be reported to and discussed with an independent Data Monitoring Committee (DMC). The Chair of the DMC will ask for review of all AEs and SAEs and an opinion will be given regarding whether they are related to the study. The results of discussions with and by the DMC will be presented to the independent Trial Steering Committee (TSC).

### Data management

All data will be handled in accordance with the 2018 Data Protection Act. Hard copies of CRFs linking patient‐identifiable information to their unique study ID and study data will be kept securely by recruiting sites and only accessible to a small number of defined people involved in the study. Anonymized data will be entered into MACRO [45]—a secure electronic data collection system, backed up to a central server—which will be developed and maintained by North Wales Organisation for Randomised Trials in Health (NWORTH) Clinical Trials Unit (CTU). MACRO has inbuilt controls to minimize data entry errors and data can be entered, reviewed and amended whilst generating an audit log. Data management is fully described in the COLO‐DETECT Data Management Plan. Data monitoring and source data verification will be conducted by the sponsor, independent of investigators, after the first 20 participants recruited at each study site and 6 monthly thereafter. All study paper and electronic data and documentation will be stored for 15 years after study completion as per sponsor and CTU policy.

### Data analysis

A full statistical analysis plan will be developed prior to analysis commencing. Statistical analysis will be performed by NWORTH CTU. The trial statistician and chief investigator will remain blinded throughout the trial and data analysis. The primary outcome, MAP in GGC versus SC, will be analysed using an ANOVA, using the randomization stratification variables as factors. The key secondary outcome, ADR, will be analysed using logistic regression, forcing trial arm into the model and including randomization stratification variables as factors. This approach will also be used to analyse the detection rate of SSPs, advanced adenomas and cancer, as well as caecal intubation rate, and effects on future colonoscopy workload. Subgroup analyses will be conducted on colonoscopist type (i.e., non‐BCSP accredited vs. BCSP accredited) and indication for colonoscopy (screening vs. symptomatic).

Whether group difference (GGC vs. SC) exists for the nature, morphology, and distribution of polyps found in the colorectum, will be analysed using a multinomial logistic regression; polyp size will be analysed using an ANOVA, as will whether group difference (GGC vs. SC) exists for withdrawal times (in procedures where no polyps were found), insertion time to the caecum (or limit of examination where the caecum is not reached), MPP and patient experience (measured using the Newcastle ENDOPREM™).

To identify changes in MAP and ADR over the time of the study (which might occur due to the trial progression effect [46]), detection rates for the first 20% of participants will be compared with those for the last 20% of participants for each colonoscopist, using repeated measures *t* tests and visual plots. Parallel data on reported detection rates amongst a selection of colonoscopists from the same units but not participating in the study will be compared with the measures obtained in the SC group.

Analyses will be performed using a 5% significance level (two‐sided) and effect size estimates will be presented with 95% confidence intervals. All analyses will be completed on an intention‐to‐treat and per protocol basis and will include all randomized patients. We will additionally undertake sensitivity analysis limiting analysis to patients with complete colonoscopy. Should substantial data be found to be missing for a measure or variable (i.e., ≥5%), predictors of missingness will be investigated using regression modelling. Multiple imputation may also be employed to address missing values where appropriate.

A ‘within study’ economic evaluation will be conducted to determine cost effectiveness of GGC versus SC from the perspective of the NHS. It will be based upon the costs of randomized interventions received and on use of subsequent care and services. Use of resources will be collected including duration of procedure, resource use and associated staff costs (including grade of staff), any additional treatment prescribed (e.g., analgesics), appointments missed, any hospital inpatient stays, and any AEs and associated treatment. The EQ‐5D‐5L will provide utility information. Incremental cost effectiveness ratio (cost per quality‐adjusted life‐year) will be estimated with robustness of results evaluated in a sensitivity analysis. The incremental cost effectiveness ratio will be compared against thresholds used to establish value for money in the NHS (currently in the region of £20 000–£30 000 per quality‐adjusted life‐year).

Economic modelling approaches will be used to explore longer‐term economic consequences and patient benefits (including effects on CRC) of GI Genius™, across a lifetime horizon.

## ETHICAL CONSIDERATIONS

COLO‐DETECT has received NHS REC (21‐WS‐0003) and Health Research Authority approval (original approval dated 25 January 2021, latest version [v3.0] approved 19 October 2021). Any further protocol modifications will be submitted for REC and Health Research Authority approval prior to implementation and communicated to all relevant bodies, including study sites and trial registries.

### Registration details

COLO‐DETECT has been prospectively registered at ClinicalTrials.gov (NCT04723758) and ISRCTN (10451355) and has been adopted to the National Institute for Health Research (NIHR) portfolio (Central Portfolio Management System ID 48022).

### Study oversight

An independent TSC will provide oversight on behalf of the sponsor and funder to ensure that the project is conducted to the rigorous standards set out in the Department of Health's Research Governance Framework for Health and Social Care [47] and the Guidelines for Good Clinical Practice [44]. The TSC will receive reports from an independent DMC which will be responsible for ‘safeguarding the interests of study participants, assessing the safety and efficacy of the interventions during the study, reviewing external evidence with an impact on risk/benefit balance and for monitoring the overall conduct of the study’ [48]. The DMC will receive reports from the Trial Management Group (TMG). The TMG will be responsible for running the study and will monitor the study including data quality and protocol compliance at all sites. Further oversight may occur in the form of evaluation by an external auditor or government inspector. All TSC and DMC committee members will complete a declaration that they have no competing interests and will inform the TMG if there are any changes to this during the course of the study.

### Dissemination

Presentations will be made to regional, national and international networks, symposia and learned scientific societies. Results will be submitted for peer‐reviewed publication in international journals. Lay summaries will be developed and disseminated to study participants, including through the website of the study team and through patient groups. A report will also be prepared by the TMG for the study funders; the funders will have no role in or authority over the preparation and publication of reports. The Chief Investigator will take responsibility to present and publish the study outcomes.

## DISCUSSION

Artificial‐intelligence‐powered CADe is an emerging and exciting field in gastrointestinal endoscopy, with the hope it will produce a step‐change in lesion detection. It follows in the footsteps of improving endoscopy technology and devices with a clear evidence base and moves from seeking further gains in mucosal visibility to focusing on improving lesion detection on visible mucosa. Internationally, many artificial‐intelligence‐based detection systems have been developed and validated, with some initial evidence from their use in clinical practice [31, 32, 33, 35, 49]. Like all new interventions and technologies, though, robust, large‐scale clinical trials set in routine practice are required to demonstrate clinical relevance and to inform changes in practice. However, adoption of novel technology into healthcare systems does not occur solely on the basis of clinical effectiveness, assessment of which should be accompanied by economic evaluation to facilitate informed commissioning and purchasing decisions.

COLO‐DETECT will help address the evidence gap by recruiting over 2000 participants (to allow for up to 10% incomplete colonoscopies) across multiple sites, including specialist and non‐specialist centres, increasing the generalizability of results. It will have the power to detect clinically significant differences in lesion detection, should they exist, by measuring any differences using MAP as primary outcome and ADR as key secondary outcome. MAP (also referred to as adenomas per colonoscopy) is an alternative measure of lesion detection that has been used in polyposis syndrome trials given the high number of polyps in this patient group. It has also been proposed as the most important marker for polyp prevention trials [50], as it gives an indication of the overall number of adenomas present, whereas ADR is only binary, that is, ≥1 or none. The use of MAP avoids the ‘one and done’ phenomenon and is coherent with the ambition to clear a colon of potentially cancerous lesions [15]. This principle is as applicable to colonoscopy in routine practice as it is to polyp prevention trials, meaning that MAP may be a more appropriate outcome measure in CADe studies than ADR [51]. However, ADR has historically been used as the main marker of colonoscopy quality. COLO‐DETECT will thus measure ADR and MAP.

Additional strengths are the prospectively planned economic evaluation, to which the secondary objectives of measuring any differences in surveillance interval and the rates of AEs will make key contributions; and a process evaluation which will seek to understand issues relevant to the implementation of this technology across clinical practice, should it prove to be clinically effective.

Furthermore, COLO‐DETECT has included comprehensive patient and participant representative involvement, along with expert independent CTU statistician input into relevant aspects of study design and analysis (for which they will remain blinded), providing further methodological rigour. The unavoidable weakness of the study is an inability to achieve participant or colonoscopist blinding due to the nature of the intervention. However, this will affect all clinical studies of this type.

In conclusion, COLO‐DETECT will study the effectiveness of a novel technology in improving detection at colonoscopy along with cost effectiveness and process issues around uptake and utilization. The results will have a significant impact on future adoption of this novel technology.

## AUTHOR CONTRIBUTIONS

AS: Trial Manager; designed protocol and wrote manuscript. LS: Co‐Chief Investigator; designed protocol and contributed to writing and review of the manuscript. JSH: contributed to protocol design and writing of the manuscript. LJN: contributed to protocol design and reviewed the manuscript. TJWL: contributed to protocol design and reviewed the manuscript. AB and RE: Clinical Trials Unit statisticians; contributed to protocol design and writing of the manuscript. LV: contributed to protocol design and reviewed the manuscript. JW will provide comprehensive patient and participant involvement representative input from the commencement of trial design and throughout. CJR: Chief Investigator; designed protocol and reviewed and contributed to writing of the manuscript.

## CONFLICT OF INTEREST

Colin J Rees has received grant funding from ARC Medical, Norgine, Medtronic, 3D Matrix Solutions and Olympus Medical. He was an expert witness for ARC Medical. The remaining authors, and all study site PIs, declare no competing interests.

## Data Availability

Data sharing is not applicable to this article as no new data were created or analyzed in this study.
